# A System for Verifying Radiation Therapy Treatments That Use Multiple Modes of Real-Time Motion Adaptation

**DOI:** 10.7759/cureus.83023

**Published:** 2025-04-26

**Authors:** Tanya Kairn, Liting Yu, Scott B Crowe

**Affiliations:** 1 Cancer Care Services, Royal Brisbane and Women's Hospital, Brisbane, AUS

**Keywords:** motion management, quality assurance, radiation therapy, radiotherapy, real-time tracking, tomotherapy

## Abstract

This technical report describes the development and use of a system for performing routine quality assurance (QA) of real-time adaptive radiotherapy treatments delivered using the Accuray Radixact Synchrony platform. Established international guidelines recommend the use of a dynamic phantom to verify real-time target tracking and the use of 2D dosimetry to verify the accuracy of modulated radiotherapy treatment delivery; hence, a dynamic QA system was developed to fulfill both of these requirements while being comparatively easy to understand, consistent with other local radiotherapy QA processes and straightforward to set up and use. The resulting dynamic QA system used a PTW Octavius 1500 ionisation chamber array dosimeter, set up in the bottom half of an Octavius II phantom, on top of a CIRS Dynamic Platform that enables superior-inferior motion, with purpose-designed 3D-printed components placed on top of the array. The 3D-printed components included lung-equivalent blocks containing a unit-density sphere, for testing Synchrony’s lung tracking with respiratory modelling mode, and five implanted gold seeds, for testing Synchrony’s fiducial tracking mode. The use of the dynamic QA system for verifying the performance of Radixact Synchrony’s fiducial tracking mode was demonstrated by measuring the dose from eight prostate treatments, while four different sample prostate motion traces were used to drive the dynamic platform. The results of this testing indicated that Synchrony was able to track the motion of the platform accurately within 1 mm (often within 0.5 mm), and local patient-specific QA gamma evaluation thresholds were consistently surpassed for all motion traces other than a high-frequency prostate motion trace. For this challenging high-frequency motion, gamma agreement indices from QA measurement results decreased by more than 3.5% when Synchrony was not used; without Synchrony’s real-time tracking, the target so often moved away from its planned location that substantial proportions of the treatments were delivered at unintended locations. The use of the dynamic QA system to verify the performance of Radixact Synchrony’s lung tracking and respiratory modelling mode was demonstrated by tracking the 3D printed unit-density sphere while the phantom moved according to a pre-programmed sinusoidal waveform. Again, the resulting 2D measurement exceeded the local patient-specific QA gamma evaluation threshold. This result, in combination with the fiducial tracking results, suggests that the dynamic QA system developed in this work would also be useful for verifying Synchrony’s performance in fiducial tracking with respiratory modelling mode, for abdominal targets. Combined testing of Radixact jaw and multileaf-collimator motions modified by Synchrony may be achieved by applying a yaw rotation to the dynamic platform so that lateral and superior-inferior phantom motions are produced. The dynamic QA system described in this technical report is well suited to routine clinical verification of tracking modes provided by Radixact Synchrony (and other real-time adaptive radiotherapy systems) during initial commissioning, periodic end-to-end testing, and routine patient-specific QA testing.

## Introduction

The need to perform two-dimensional (2D) dose verification measurements is a long-established aspect of quality assurance (QA) for modulated radiotherapy techniques, such as dynamic conformal arc therapy (DCAT), intensity-modulated radiotherapy (IMRT), volumetric arc radiotherapy (VMAT), or helical tomotherapy [1-5]. For helical tomotherapy, in particular, the American Association of Physicists in Medicine (AAPM) has recently highlighted the need to extend system commissioning and patient-specific QA processes to include monthly delivery of treatment plans to moving phantoms, to test all available real-time target tracking modes [6].

The Accuray Radixact helical tomotherapy system incorporates Synchrony motion management (Accuray Inc., Sunnyvale, CA), with real-time target tracking achieved by repositioning of the jaws and multi-leaf collimator leaves in response to changes in target position [6]. The Synchrony system uses frequent kV imaging (up to one image every two seconds, if averaged over the full 360 degree gantry rotation, at the fastest gantry speed) to build target motion models based on the positions of implanted fiducial markers alone, or based on a combination of the target position and an external respiratory surrogate, with or without implanted fiducials [6,7].

Radixact Synchrony software and documentation specify three modes of motion tracking: “Synchrony Fiducial Tracking”, “Synchrony Fiducial Tracking with Respiratory Modelling”, and “Synchrony Lung Tracking with Respiratory Modelling” [7]. The latter two modes use LED markers placed on the patient’s chest or abdomen that track surface motion, in combination with kV images that track the internal motion of either fiducial markers (fiducial tracking) or a clearly defined lung target (lung tracking). Respiratory modes each build a predictive model of the patient’s respiration that is continually verified throughout treatment and rebuilt when necessary, with reference to user-defined tolerances [7].

The Synchrony fiducial tracking mode uses kV imaging to track the internal non-respiratory motion of fiducial markers without a predictive model. In fiducial tracking mode, Synchrony uses the term “model” to refer to the 3D position of the target calculated from successive kV images. Two sequential images are required to calculate this position, and the position is updated when two sequential images show that the current model is incorrect [7].

Generally, fiducial-only tracking with a quasi-static model is used for non-respiratory motion for targets such as the prostate, whereas the respiratory modelling modes are used for respiratory motion, without fiducials for high-contrast targets such as lung tumors and with fiducials for low-contrast targets such as in the abdomen.

Commercial phantoms with the potential to verify Synchrony respiratory tracking include the QUASAR Respiratory Motion Phantom [8] and the CIRS Dynamic Thorax Phantom [9]. These allow motion to be driven by specified waveforms, including patient motion traces, and facilitate 2D dose measurements using small pieces of radiochromic film placed within the moving component of each phantom. The heterogeneous and broadly humanoid geometry of these phantoms is clearly advantageous for commissioning and end-to-end testing, when compared to the homogeneous phantom designs that are often used for radiotherapy QA measurements [3,9]. However, the utility of these humanoid phantoms for routine use is restricted by the limitation that 2D dosimetry can only be achieved with film placed in a specific location, rather than with electronic array dosimeters that produce 2D dose measurements across a comparatively large area and provide immediate readout of results [4,9].

The ideal 2D dosimetry system for verifying the performance of the Radixact Synchrony system would therefore accommodate an electronic array dosimeter in a setup that would allow programmable motion, with heterogeneous components, including a lung target and fiducials for testing Synchrony’s motion adaptation modes. Preferably, the system would also be easy to understand, consistent with other local radiotherapy QA processes, and straightforward to set up and use, to minimise interruption of the radiotherapy treatment preparation process. This technical report describes the development and use of a system that fulfils these requirements, especially for superior-motion, for performing routine QA of jaw tracking aspects of Radixact Synchrony treatment delivery.

## Technical report

Development of the dynamic QA system

The Radixact treatment unit is supplied with a physics testing package that can include a CIRS Dynamic Platform (Sun Nuclear Corporation, Melbourne, FL), a PTW Octavius II phantom, and a PTW Octavius 1500 ionisation chamber array (PTW Freiburg GmbH, Germany). Previous studies have demonstrated the value of placing array dosimeters on moving platforms to evaluate the accuracy of modulated radiotherapy treatments in the presence of motion and motion interplay [10-12]. Accordingly, the first step in the development of a routine dynamic QA system for Radixact Synchrony was to identify a method by which the Octavius 1500 array could be used with the dynamic platform.

The CIRS Dynamic Platform allows up to 5 cm of motion in the superior-inferior direction, using built-in waveforms or imported respiratory trajectory data, with the potential to deliver up to 5 cm of anterior-posterior motion of an external surrogate, if needed. The PTW Octavius 1500 detector is an array of 1,405 plane-parallel vented ionisation chambers, with 0.7 cm detector spacing and a total active area of 27 x 27 cm^2^. A non-standard setup was devised to position the Octavius 1500 array on the dynamic platform.

The PTW Octavius II phantom is an approximately water-equivalent (1.04 g/cm^3^) octagonal prism made from polystyrene. Because the 27 kg weight of the Octavius II phantom was close to the 32 kg weight limit of the CIRS Dynamic Platform, only the bottom half of the phantom was used in this study to ensure that the quoted 0.1 mm positioning of the CIRS Dynamic Platform could be achieved with maximum precision. Similarly, to avoid collision between the electronics at the inferior end of the Octavius 1500 array and the drive system of the dynamic platform, the Octavius II phantom and the Octavius 1500 array were set up for CT scanning and treatment delivery using a feet-first orientation, with the smiling side of the Octavius II phantom facing towards the gantry. Accordingly, all planned dose distributions needed to be rotated by 180 degrees in the accompanying PTW Verisoft QA analysis software, prior to comparison with each corresponding Octavius 1500 measurement.

To allow the dynamic QA system to reliably test the performance of the Radixact Synchrony system, a novel insert was designed and 3D-printed [13] to fit into a pre-existing “inhomogeneity insert” for the Octavius II phantom. A Raise 3D Pro 2 printer (Raise 3D Technologies Inc., Irvine, CA) was used to print three 15 x 5 x 2 cm^3^ blocks in low-density foaming polylactic acid filament (PLA-LW, eSUN Industrial Co., Shenzhen, China) to achieve a lung-equivalent density using a method described by Crowe et al. [14]. One of the printed blocks contained a spherical void to accommodate a unit-density 1.5 cm diameter sphere that was printed in a standard polylactic acid filament using the same printer. This sphere was used as the lung target for Synchrony lung tracking with respiratory modelling mode verification.

To enable Synchrony fiducial tracking tests using prostate treatment plans, five gold seed markers (QLRAD B.V., Almere, Netherlands) were implanted into the lung-equivalent 3D print using a needle and obturator. Care was taken to avoid alignment in likely imaging directions when placing the seeds.

**Figure 1:**
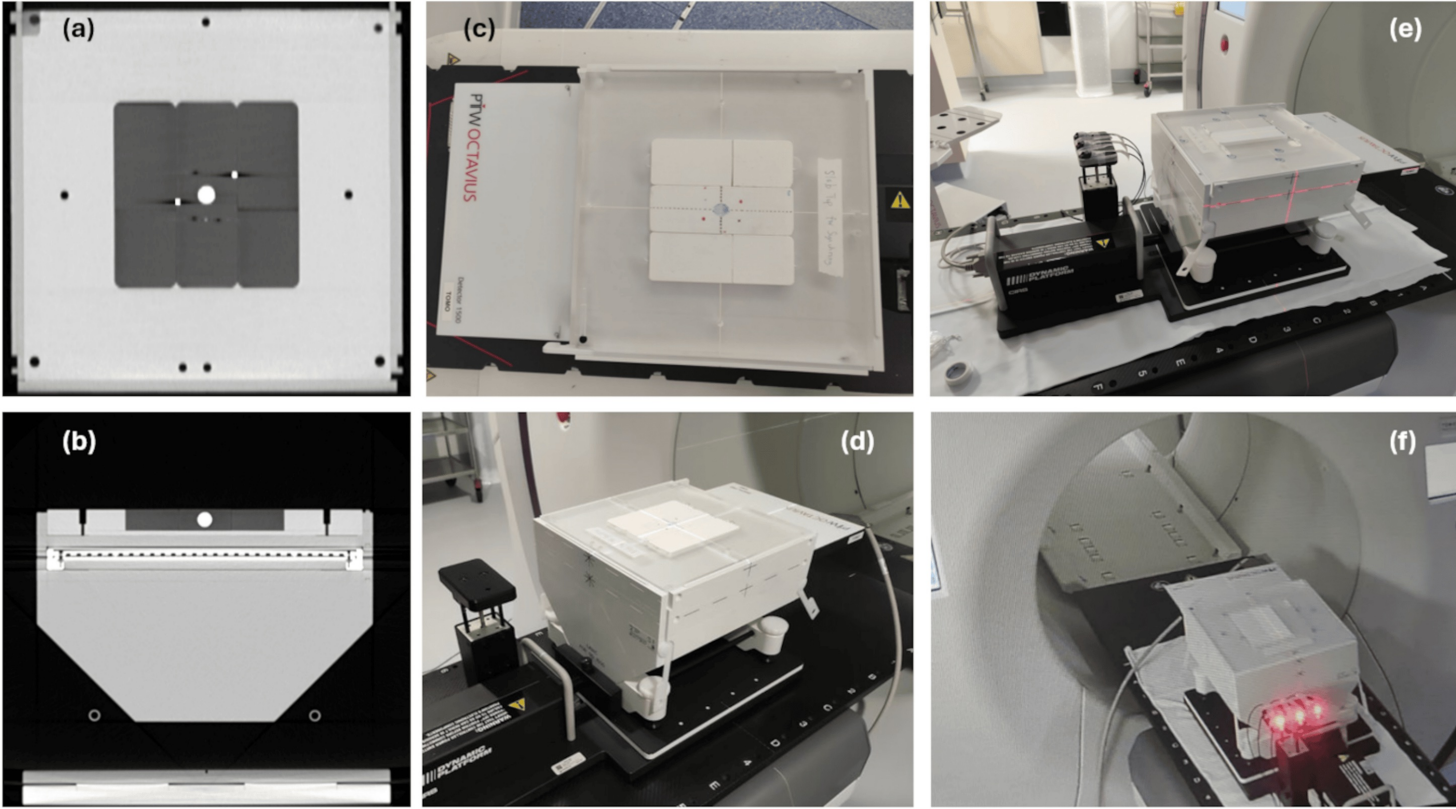
CT images and photographs of the dynamic QA system setup for Synchrony verification. Dynamic QA system as shown in (a) coronal and (b) transverse slices through the CT scan, (c) and (d) photographs of the Synchrony fiducial tracking verification setup, and (e) and (f) photographs of Synchrony lung tracking with the respiratory modelling verification setup.

Figure 1 shows pictures of the dynamic QA system that was devised for Synchrony verification. The Octavius 1500 array sits in the bottom half of the Octavius II phantom, on the dynamic platform, with the inhomogeneity insert containing lung-equivalent 3D-printed blocks, target volume, and fiducial markers, placed over the Octavius 1500 array. Informative images from CT scans of the combined phantom are also included in this figure to illustrate the density of the dynamic platform and the 3D-printed components in comparison to the densities of the Octavius II phantom and the Octavius 1500 detector.

Note that, for CT scanning, the bottom part of the Octavius II was replaced with a solid section designed specifically for dose calculation, whereas, for treatment delivery, the bottom part of the Octavius II phantom, containing the semicircular air gap that corrects for array dose response from posterior angles of incidence [3] was used. Other than the air gap, the geometries and densities of these two phantom components were identical.

Use of the dynamic QA system: treatment planning

All treatment plans were generated using the Accuray Precision treatment planning system (Accuray Inc., Sunnyvale, CA). For the purpose of Radixact Synchrony verification, a QA template plan was created using a feet-first CT scan of the dynamic QA system described in the previous section. The template plan creation process included replacing the CT couch, setting up a mock treatment with arbitrary parameters, selecting the Synchrony mode, and selecting kV imaging angles.

For Radixact, imaging frequency is determined by the proximity of the gantry angles at which imaging is chosen to occur each time the gantry rotates during the helical treatment delivery. Variable imaging frequency is achieved by selecting gantry angles that are closer together around anatomical regions of interest or further apart to avoid imaging through high-density anatomy or implants. While imaging angles can be adjusted on-the-fly by users during Radixact Synchrony treatment delivery, imaging angles were kept fixed for this study. Six equidistant gantry angles (0, 60, 120, 150, 180, 240, and 300 degrees) were selected for imaging during these Synchrony QA deliveries.

Patient-specific QA plans were created in Precision using the template plan, by mapping the centre of each treatment target onto the central ionisation chamber in the Octavius 1500 array and then completing a high-resolution dose calculation, defining each resulting QA plan as “deliverable” by Radixact, and exporting each dose distribution and QA treatment plan for treatment delivery.

Exported data were then handled by a preexisting in-house daemon script, which routinely listened for DICOM exports that arrived at a specified network location and then moved the exported treatment plans and dose files into appropriately named directories for each patient, and produced patient-specific QA report forms ready for completion after QA treatment delivery.

Use of the dynamic QA system: lung tracking with respiratory modelling mode verification

The dynamic QA system described in the previous sections was ultimately put into clinical use for QA of Synchrony treatments that used fiducial tracking mode (see next section). However, a simple proof-of-concept measurement was also completed to demonstrate the use of the system in verifying a treatment that used Synchrony’s lung tracking with the respiratory modelling mode.

A conventionally fractionated lung treatment of 60 Gy in 30 fractions for a planning target volume of 170 cm^3^ was selected for use in this demonstration. The Radixact treatment plan used a gantry period of 22.2 s, resulting in a 3.70 s period between acquiring each of the six Synchrony kV images per gantry rotation. The Radixact dynamic jaw was used for this treatment, with a planned maximum jaw size of 25 mm.

The treatment had a planned beam-on time of 3.4 minutes, requiring 9.2 gantry rotations and at least 55 kV images for Synchrony lung tracking. The Octavius 1500 measurement was started as soon as the treatment was prepared for delivery and not stopped until after the treatment console indicated that the treatment was complete. Consequently, the full imaging dose from all of the Synchrony kV images (at least 0.4 cGy) was included in the QA measurement.

The QA treatment plan was generated, as described in the previous section, with the small 3D-printed sphere contoured and designated as the internal lung target for Synchrony to track. The dynamic QA system was set up to use the external surrogate platform on the CIRS Dynamic Platform with the Synchrony LED surface markers, as shown in Figure 1(e) and Figure 1(f).

Rather than using a patient’s respiratory trace, this simple test used a pre-programmed sinusoidal waveform to drive the CIRS Dynamic Platform. A superior-inferior amplitude of 10 mm was selected for the platform, mimicking the amplitude of typical lung target motions [15]. The external surrogate platform was driven with a reduced anterior-posterior motion amplitude of 5 mm to account for the reduced motion in this direction that is frequently observed clinically.

Synchrony settings used during treatment delivery were evaluated for potential use as clinical tolerances: a potential difference threshold of 2 mm, a measured delta threshold of 4 mm, and a target offset threshold of 30 mm.

**Figure 2:**
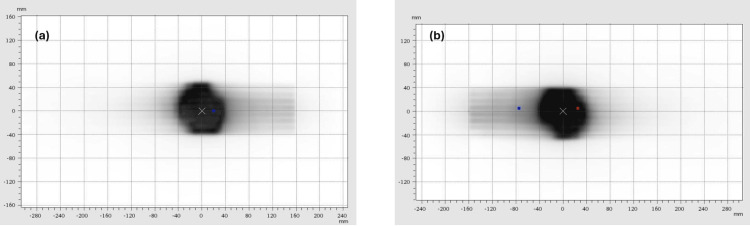
Verifying dose delivery: lung cancer treatment with and without motion. Illustrations of 2D dose measurements from the Octavius 1500 detector used (a) in the dynamic QA system, with Synchrony used to track and correct for sinusoidal target motion, and (b) in a conventional QA setup, in the full Octavius II phantom, without the heterogeneous insert, without phantom motion. Both images are overlaid with gamma evaluation results: red points indicate that the measured dose was greater than the planned dose and blue points indicate that the measured dose was less than the planned dose. Note that, due to the feet-first orientation of the dynamic QA phantom, the dynamic result in (a) is rotated 180 degrees relative to the static result in (b).

Figure 2 shows the result of using the Radixact Synchrony system to achieve real-time adaptation to the movement of the dynamic QA system (Figure 2(a)), in comparison to the result of performing a measurement of the same treatment delivered to the standard Octavius II phantom without motion. For both measurements, gamma evaluations were completed using our standard criteria, 2% and 2 mm, with a 5% low dose threshold [16,17].

The Octavius 1500 measurements for this case achieved passing gamma evaluation results, both exceeding 99%, indicating close agreement between the planned and measured 2D dose distributions. When Synchrony was used to account for the motion of the dynamic QA system, the resulting gamma agreement index was 99.8%. Performing this QA test using a more conventional setup with no phantom motion at all gave a gamma evaluation index of 99.6%.

The strong similarity between the two results shown in Figure 2, where the measurement made using the static phantom included no imaging dose and the Synchrony measurement included dose from at least 55 images, confirms previous observations of the minimal contribution that Synchrony kV imaging makes to soft-tissue (or plastic phantom) dose [18].

Use of dynamic QA system: routine QA of fiducial tracking mode

The dynamic QA system developed in this work has been used routinely for patient-specific QA of the early Radixact Synchrony prostate fiducial tracking cases treated in our facility. The eight cases evaluated for this report were all treated in the first five months after prostate fiducial marker tracking was clinically released in our facility.

Three of the treatments were planned as phase two boosts to the prostate plus proximal seminal vesicles (SV), with the remaining five treatments being planned for the prostate only; no nodes were included in any of these treatments. Three of the prostate-only treatments were delivered using 3 Gy fractional doses, and all of the other treatments were delivered using 2 Gy per fraction. Given this conventional fractionation, the 2D dose measurement results produced by this work were all tested for agreement with their corresponding planned dose distributions using 2%/2 mm gamma evaluation criteria according to local practice [16,17], rather than the 3%/1 mm criteria that are increasingly used for stereotactic body radiotherapy or the looser 3%/3 mm criteria that are recommended by the AAPM for evaluating helical tomotherapy treatments with real-time adaptation [6]. By local convention, gamma agreement indices of 95% or more were used to define passing QA results [16,17].

The Radixact dynamic jaw was used for all of these treatments, with a planned maximum jaw size of 25 mm. Planned beam-on times ranged from 2.8 to 4.8 minutes, and the planned gantry periods were in the range 17.7-20.1 seconds, except for one outlier with a gantry period of 15.3 seconds. Accordingly, the six equidistant kV images were planned to be delivered with a period of 2.95-3.35 seconds, except for the outlier, who was imaged every 2.55 seconds.

Synchrony settings used during treatment delivery were selected to match clinical tolerances used locally: a potential difference threshold of 3 mm, a rigid body threshold of 1.5 mm, and a target offset threshold of 30 mm. A tracking range of 20 mm was used, with medium sensitivity and an auto-pause delay of 20 seconds.

Synchrony’s prostate fiducial tracking capability was tested by driving the dynamic QA system using four different prostate motion traces that had been used in previous studies of motion adaptation systems [15,19,20]. The four traces were characterized as stable prostate motion, continuous drift, erratic prostate motion, and high-frequency prostate motion [15]. No external surrogate motion was needed, since the Synchrony fiducial tracking mode does not use the LED surface markers, and so no motion was applied to the surrogate motion element of the dynamic platform.

**Figure 3:**
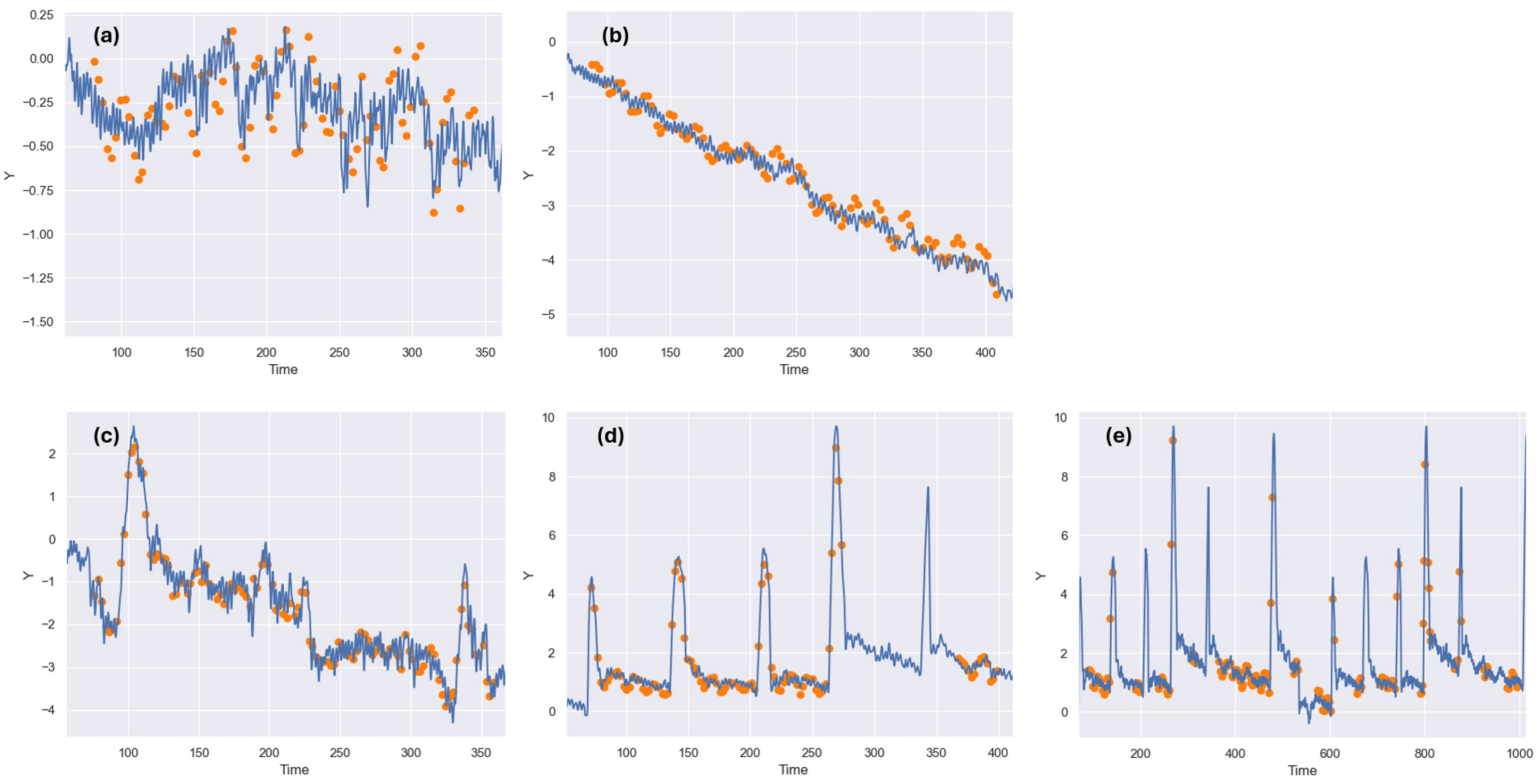
Verification of Synchrony’s identification of fiducial motion trajectories. Superior-inferior fiducial motion detected by Synchrony during prostate treatments delivered to dynamic QA phantom (orange dots) compared to programmed motion delivered by dynamic platform (blue line), during (a) stable prostate motion, (b) continuous drift, (c) erratic prostate motion, and (d) and (e) high-frequency prostate motion for two different treatments. All horizontal scales show time in seconds and all vertical scales show distance in mm.

Patient-specific QA tests were performed for eight Synchrony prostate treatments by repeatedly delivering all treatments with no motion and with each of the four motion traces and comparing the resulting 2D measurements against each corresponding planned treatment dose. The Accuray MotionQA tool allowed the fiducial motion observed by Synchrony to be extracted and compared against the programmed motion for each of these treatment deliveries, producing results that are exemplified in Figure 3.

The data shown in Figure 3 are reasonably representative of results for all the measured treatment plans. Visual examination of plots such as the five shown in Figure 3 indicated that, for all eight treatments delivered with all four prostate motion traces, Synchrony’s detected positions of the fiducials in the dynamic QA phantom were consistently within 1 mm (and often within 0.5 mm) of their programmed positions.

The number of occasions where Synchrony paused treatment to obtain additional kV images and rebuild its synchronization model was recorded for each treatment delivery. No pauses were observed when stable prostate motion and continuous drift motion traces were used to drive the dynamic QA system. No more than two (and usually zero) treatment pauses occurred when the erratic prostate motion trace was used.

By contrast, the high-frequency prostate motion trace provided an especially challenging test for Synchrony, with all treatments being paused at least once. One treatment was paused five times, and two treatments were paused six times, requiring multiple attempts for Synchrony to rebuild its motion model. Treatment pauses are visible in the result shown in Figure 3(d) and Figure 3(e), as regions where gaps appear in the series of dots representing the phantom position detected by Synchrony, while the line representing the programmed phantom motion continues unbroken.

The gantry period used for treatment shown in Figure 3(d) was appreciably shorter than the gantry period used for the treatment shown in Figure 3(e), producing a correspondingly shorter imaging period with multiple images acquired each time the respiratory trace jumped away from its baseline position. The treatment in Figure 3(d) was completed with only one treatment pause, even though the dynamic QA system moved with the same trajectory as the treatment that paused multiple times in Figure 3(e). Note also that, although the planned beam-on time for these prostate treatments had an average of four minutes and a maximum of 4.8 minutes, the treatment shown in Figure 3(e) took more than 15 minutes to complete due to the duration of the multiple pauses needed for Synchrony to achieve a reliable tracking model.

**Figure 4:**
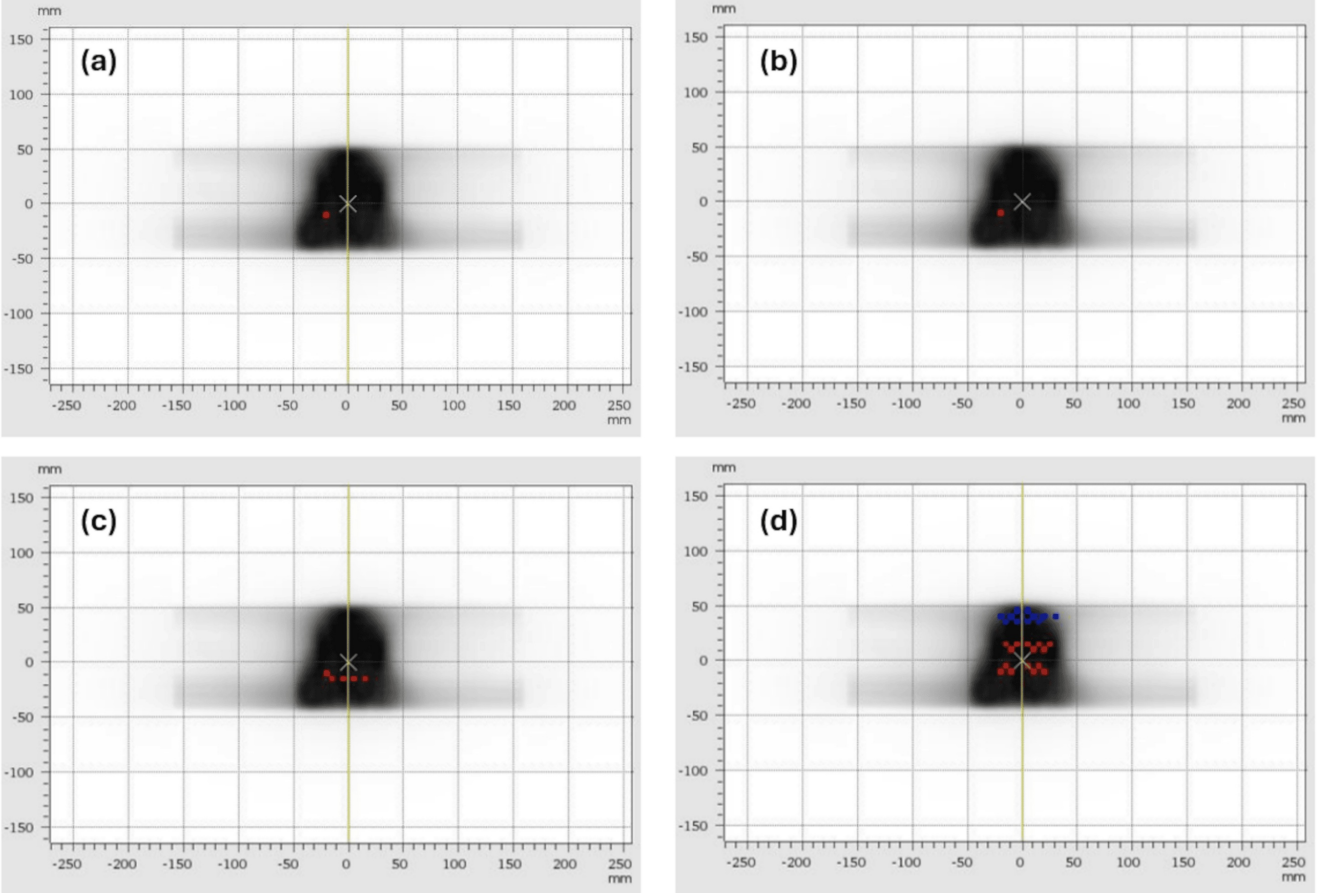
Verifying dose delivery: comparatively long prostate treatment (4.4 min planned beam-on time). Illustrations of 2D dose measurements from the Octavius 1500 detector used in the dynamic QA system, with overlying gamma evaluation results: red points indicate that the measured dose was greater than the planned dose and blue points indicate that the measured dose was less than the planned dose. Measurements were made during (a) stable prostate motion, (b) continuous drift, (c) erratic prostate motion, and (d) high-frequency prostate motion. Note that, due to the feet-first orientation of the phantom, the superior end of the prostate appears at the inferior end of each image, and vice versa.

**Figure 5:**
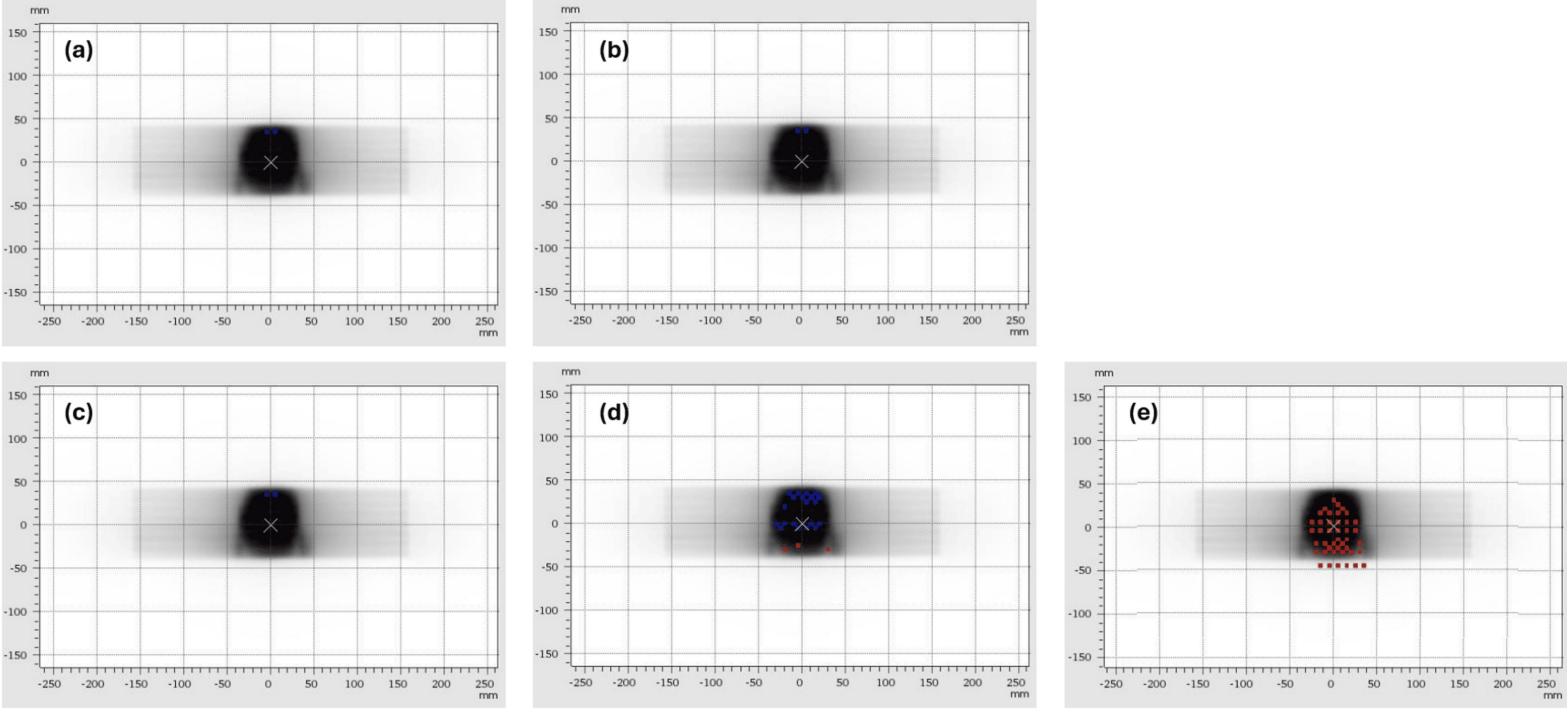
Verifying dose delivery: comparatively short prostate treatment (2.8 min planned beam-on time). Illustrations of 2D dose measurements from the Octavius 1500 detector used in the dynamic QA system, with overlying gamma evaluation results: red points indicate that the measured dose was greater than the planned dose and blue points indicate that the measured dose was less than the planned dose. Measurements were made during (a) stable prostate motion, (b) continuous drift, (c) erratic prostate motion and (d) high-frequency prostate motion. The measurement shown in (e) was completed without using Synchrony, with high-frequency prostate motion. Note that due to the feet-first orientation of the phantom, the superior end of the prostate appears at the inferior end of each image, and vice versa.

The results of the patient-specific QA measurements completed for the eight treatment plans using the four different prostate motion traces are shown in Figures 4-5. Figure 4 shows a typical set of results for one of the larger prostates, with all four measurements achieving gamma agreement indices over 95% and therefore passing our local QA test. This result was consistent between all eight prostate treatment plans tested, with all plans achieving gamma agreement indices above 99% with Synchrony tracking of stable, continuous drift and erratic prostate motions.

The obvious exception was the high-frequency prostate motion trace, which produced a technically passing result (95.2%) for the treatment shown in Figure 4(d), but which produced failing results (90.5%, 92.9%, and 92.4%) in three of the remaining seven treatments.

For comparison, Figure 5 shows a typical set of QA results for one of the smaller prostates. Again, the gamma agreement indices were all above 99% when Synchrony was used with the dynamic QA system programmed to produce stable, continuous drift and erratic prostate motion, while the gamma agreement index was noticeably lower (95.3%) when the high-frequency prostate motion trace was used.

Figure 5 also includes an indication of the effect of delivering these treatments to the moving phantom without allowing the Radixact system to compensate for the motion using Synchrony. An example result of not using Radixact Synchrony real-time fiducial tracking is shown in Figure 5(e), for the same treatment and high-frequency motion trace that is shown in the Synchrony result in Figure 5(d). The QA measurement that was completed without Synchrony in Figure 5(e) produced an unequivocally failing result (91.7%).

Use of the dynamic QA system: numerical data

For reference, Table 1 provides numerical data describing the methods and results from this technical report, including the treatment target, prescription, and plan data, as well as QA measurement results and, in parenthesis, counts of the number of treatment pauses and failures to rebuild the Synchrony model that occurred during each QA delivery. The planned gantry period (number of seconds taken by each gantry rotation) and the planned imaging period (number of seconds between each Synchrony kV image acquisition) are also included, indicating how for Radixact Synchrony imaging frequency is determined by gantry speed.

**Table 1:** Summary of treatment plan parameters and QA measurement results. Relevant QA treatment plan parameters summarised for comparison with gamma agreement indices (GAI) resulting from measurements made using the dynamic QA system. Results are shown as (percentage GAI) ((number of times Synchrony was unable to rebuild the synchronization model) / (number of times Synchrony paused the treatment)). To clarify discussion, prostate cases are listed in order of increasing imaging period, rather than in order of treatment delivery.

Patient ID (arbitrary)	00	01	02	03	04	05	06	07	08
Treatment site	Lung	Prostate	Prostate & SV (Ph2)	Prostate	Prostate	Prostate	Prostate	Prostate & SV (Ph2)	Prostate & SV (Ph2)
Dose per fraction (Gy)	2.0	2.0	2.0	3.0	3.0	3.0	2.0	2.0	2.0
Number of fractions planned	30.0	39	15.0	20.0	20.0	20.0	39	16.0	16.0
Planned beam-on time (min)	3.4	4.8	4.4	4.1	4.0	3.9	2.8	3.1	4.0
Planned beam-on time (s)	203.7	285.7	263.7	246.7	242.2	235.9	170.3	184.7	239.5
Gantry period (s)	22.2	15.3	17.7	19.5	19.7	19.9	20.1	20.1	20.1
Imaging period (s)	3.70	2.55	2.95	3.25	3.28	3.32	3.35	3.35	3.35
Number of Synchrony imaging angles	6.0	6.0	6.0	6.0	6.0	6.0	6.0	6.0	6.0
Number of gantry rotations per fraction	9.2	18.7	14.9	12.7	12.3	11.9	8.5	9.2	11.9
Min number of Synchrony images per fraction	55	112	89	76	74	71	51	55	71
Min Synchrony imaging dose in QA measurement (cGy)	0.40	0.81	0.64	0.55	0.53	0.51	0.37	0.40	0.51
Imaging angle separation (°)	60	60	60	60	60	60	60	60	60
Nominal jaw separation (mm)	25	25	25	25	25	25	25	25	25
Helical tomotherapy pitch	0.442	0.233	0.303	0.295	0.295	0.233	0.446	0.303	0.233
GAI, standard Octavius II setup, no motion	99.6	100.0	100.0	100.0	100.0	100.0	100.0	99.5	100.0
GAI, dynamic QA system, sinusoidal motion	99.8 (0/0)	-	-	-	-	-	-	-	-
GAI, dynamic QA system, no motion	-	99.8 (0/0)	99.8 (0/0)	99.6 (0/0)	99.6 (0/0)	100 (0/0)	100 (0/0)	99.8 (0/0)	99.8 (0/0)
GAI, dynamic QA system, stable motion	-	99.8 (0/0)	99.8 (0/0)	99.8 (0/0)	99.6 (0/0)	100 (0/0)	99.6 (0/0)	99.8 (0/0)	99.6 (0/0)
GAI, dynamic QA system, continuous drift motion	-	99.8 (0/0)	99.8 (0/0)	100 (0/0)	99.6 (0/0)	99.8 (0/0)	99.6 (0/0)	99.8 (0/0)	99.8 (0/0)
GAI, dynamic QA system, erratic motion	-	99.6 (0/0)	99.1 (0/0)	99.8 (0/1)	99.2 (0/2)	99.0 (0/2)	99.6 (0/1)	99.8 (0/2)	99.4 (0/0)
GAI, dynamic QA system, high frequency motion	-	97.2 (0/1)	95.3 (0/1)	90.5 (3/5)	92.4 (2/6)	95.5 (1/6)	95.2 (1/2)	92.9 (2/5)	99.4 (0/1)

## Discussion

The dynamic QA system developed through this work uses a phantom that is comparatively simple compared to the range of densities and geometries that occur in real radiotherapy patients. However, this system has obvious advantages in terms of its ease of use (including speed of setup) and compatibility with other QA systems, especially in a facility where PTW Octavius phantoms and detectors were already in use. Key characteristics of the dynamic QA system that enable the testing of multiple modes of target tracking for Radixact Synchrony in particular are the inclusion of a small spherical target within lung-equivalent materials, for investigating Synchrony lung tracking with respiratory modelling (see Figure 2), as well as several gold seed markers, for investigating Synchrony fiducial tracking (see Figures 4-5). The results of the lung tracking with respiratory modelling QA test, as well as the multiple fiducial tracking QA shown in this technical report, suggest that this dynamic QA system would also be useful for verifying Synchrony’s performance in fiducial tracking plus respiratory modelling mode, for treatments such as liver or kidney or other anatomical sites.

This dynamic QA system can be used to quickly and easily fulfill the commissioning, QA, and end-to-end requirements for motion tracking helical tomotherapy systems recommended by the AAPM [6] while providing the 2D dose measurements that are recommended across many forms of modulated radiotherapy [1,2]. The key limitation of the dynamic QA system developed in this work is that it is based on a dynamic platform that moves longitudinally along the treatment couch, moving the phantom only in the superior-inferior direction and therefore testing motion adaptation using the Radixact jaws only, and not the multi-leaf collimator. This limitation could be avoided through the purchase of a more sophisticated platform or robot that could move the dosimeter in three dimensions [10,19]. However, a less costly solution would be to simply apply a yaw rotation to the dynamic platform while keeping the Octavius phantom set up parallel to the couch as usual. This arrangement would allow the Octavius 1500 panel to produce a 2D measurement that is directly comparable to the planned dose, while motion is applied diagonally rather than longitudinally, forcing all of the Radixact unit’s collimation systems to work in concert via Synchrony, to keep the beam directed towards the intended parts of the phantom throughout treatment delivery. Production of a simple alignment jig could allow this more complicated arrangement to be set up and used without unacceptably delaying the performance of routine Synchrony QA tests.

The effects of varying the Synchrony imaging period were not explicitly evaluated in this study. However, Table 1 lists several different imaging periods that arose from the innate variation of the gantry period in these different treatment plans. For the prostate treatments that were measured while driving the dynamic QA system using the high-frequency prostate motion trace, it is apparent from data listed in Table 1 that imaging periods greater than 3 seconds were used in all of the cases where Synchrony paused the treatment mode than once, all of the cases where the number if occasions where Synchrony was unable to rebuild the synchronization model more than twice, and all of the cases where the QA measurement failed to reach the 95% passing threshold.

Results exemplified in Figure 3(d) and Figure 3(e) indicated that the Synchrony fiducial tracking system was able to accurately identify the location of the fiducials throughout the treatments to the dynamic QA system, even when using the high-frequency prostate motion trace. However, since the Radixact Synchrony fiducial tracking mode requires two sequential images to verify or adjust the 3D position of the target, increasing the imaging period leads to the potential to miss or misidentify the brief position jumps seen in the high-frequency prostate motion trace.

Evidently, this high-frequency prostate motion trace acted as a challenging stress test for the Synchrony system. In the unlikely event that a patient presents for treatment with prostate motion as irregular as is found in the high-frequency prostate motion trace [15,20], it may be advisable to delay treatment while their digestion settles. It is also worth noting that, without the use of Synchrony to provide some adaptation to this challenging motion, the QA measurement results were dramatically worse (for example, see Figure 5(d) compared to Figure 5(e)). Without Synchrony real-time tracking, the target so often moved away from its planned location that substantial proportions of the treatments were delivered at unintended locations.

The QA results achieved when using Synchrony in lung tracking with respiratory modelling mode for a simple sinusoidal motion (see Figure 2) and using Synchrony in fiducial tracking for the more clinically ealistic prostate motion traces (stable motion, continuous drift, and erratic motion; for examples, see panels (a), (b), and (c) in Figure 4 and Figure 5). This indicates that, despite simultaneous phantom, jaw, and couch motion, the use of the Synchrony system enabled all of the treatment dose distributions to be delivered to the dynamic QA phantom as though the phantom were not moving at all. For these treatments, all dose segments reached the parts of the phantom that they were planned to reach, with negligible differences (all gamma agreement indices equal to 100%) between measurements made with and without the phantom motion.

The strong agreement observed between all measurements made with the dynamic QA system and the corresponding planned dose distributions for all scenarios other than the high-frequency prostate motion scenario (see Table 1) should also be considered with an appreciation that the Octavius 1500 measurements encompassed all of the imaging dose from the large numbers of kV images used by the Synchrony system throughout each QA treatment delivery. The minimum number of kV images acquired during each treatment delivery listed in Table 1 ranges from 51 to 112, with corresponding minimum imaging doses of 0.37-0.81 cGy, although these numbers do not include images acquired (and measured) during initial model building and during treatment pauses. By comparison, the QA results obtained without motion, also listed in Table 1, included no imaging dose at all. The consistency of results made with motion (other than high-frequency prostate motion, discussed above) and without motion suggests that Radixact Synchrony imaging dose is low enough to have a negligible effect on measured treatment dose, at least for an ionization chamber array. A comprehensive evaluation of Radixact Synchrony imaging dose has previously been completed by Ferris et al. 18], similarly showing minimal imaging dose to soft tissue from acquiring one hundred Radixact Synchrony kV images per treatment.

## Conclusions

In this technical report, the development and use of a dynamic QA system capable of programmed motion in the superior-inferior direction showed that the Radixact Synchrony system was able to track complex fiducial motions and cyclical lung target motions consistently, with an accuracy within 1 mm (often within 0.5 mm). In lung tracking and respiratory modelling mode and in fiducial tracking mode, the use of the Synchrony system produced QA measurement results in close agreement with planned (greater than 99% gamma agreement indices), except when a high-frequency prostate motion trace was used to drive the dynamic QA phantom. Taken together, these results suggest that the dynamic QA system developed in this work would also be useful for verifying Synchrony’s performance in fiducial tracking with respiratory modelling mode, for abdominal targets. Combined testing of Radixact jaw and multileaf-collimator adjustments may be achieved by applying a yaw rotation to the dynamic platform to produce lateral, as well as superior-inferior phantom motions for Synchrony verification.

Overall, the dynamic QA system described in this technical report is well suited to routine clinical verification of tracking modes provided by Radixact Synchrony (and other real-time adaptive radiotherapy systems) during initial commissioning, periodic end-to-end testing, and routine patient-specific QA testing.
